# Hemostatic Effect of Oxidized Regenerated Cellulose in Simultaneous Bilateral Total Knee Arthroplasty: A Retrospective Study

**DOI:** 10.7759/cureus.94414

**Published:** 2025-10-12

**Authors:** Atsushi Sato, Kensuke Sasaki, Marika Mukunoki, Kanako Izukashi, Masataka Ota, Takayuki Okumo, Takayuki Koya, Koji Kanzaki

**Affiliations:** 1 Orthopaedic Surgery, Showa Medical University Fujigaoka Hospital, Yokohama, JPN; 2 Physiology, Showa Medical University Graduate School of Medicine, Tokyo, JPN; 3 Orthopaedic Surgery, Showa Medical University Koto Toyosu Hospital, Tokyo, JPN

**Keywords:** bilateral surgery, blood loss, hemostasis, oxidized regenerated cellulose, total knee arthroplasty

## Abstract

Background

Oxidized regenerated cellulose (ORC) is a biodegradable hemostatic agent widely used in surgical practice. However, its efficacy and safety in total knee arthroplasty (TKA), particularly in simultaneous bilateral procedures, remain underreported.

Methods

This retrospective study included patients who underwent simultaneous bilateral TKA at our institution. A total of 49 patients were analyzed and divided into two groups: the ORC group (n=21), in which ORC was used intraoperatively, and the non-ORC group (n=28). Perioperative hemoglobin (Hb) and hematocrit (Ht) levels were compared between groups, along with estimated blood loss (EBL) on postoperative days 4 and 7, calculated using the Gross formula. Transfusion rates and postoperative complications were also evaluated. Statistical analyses were performed using the Wilcoxon rank-sum test and chi-squared test.

Results

No significant preoperative differences in Hb or Ht values were observed between groups. However, both postoperative Hb/Ht levels and EBL on postoperative days 4 and 7 were significantly lower in the ORC group (p<0.05). No significant difference in transfusion rate was found between groups. Importantly, no postoperative complications directly related to ORC use were identified.

Conclusion

ORC demonstrated effective and safe hemostasis in simultaneous bilateral TKA, contributing to reduced postoperative blood loss without increasing complications. ORC may be a valuable adjunct for blood loss management in TKA.

## Introduction

Oxidized regenerated cellulose (ORC) is a biodegradable and bioabsorbable hemostatic material that promotes clot formation by providing a physical matrix for platelet aggregation and inducing local vasoconstriction through its acidic environment [1]. While extensively used in cardiovascular, thoracic, and gynecological surgeries, its application in orthopedic procedures, particularly total knee arthroplasty (TKA), remains limited. Recent randomized controlled trials (RCTs) have demonstrated that intra-articular application of ORC in unilateral TKA significantly reduces both total and hidden blood loss without increasing complication rates [1]. Furthermore, ORC has shown hemostatic efficacy comparable to topical tranexamic acid (TXA), which is widely regarded as the gold standard for perioperative blood conservation [2].

Although TXA has a well-established role in TKA blood management [3], its use is contraindicated in certain patients, such as those with a history of thromboembolic events, seizure disorders, or hypersensitivity reactions [4]. In such cases, mechanical agents like ORC may serve as a practical alternative or adjunct to pharmacologic therapy.

Simultaneous bilateral TKA (Sim B-TKA) is increasingly performed in patients with advanced bilateral knee osteoarthritis, offering advantages in efficiency and rehabilitation. However, compared with unilateral procedures, Sim B-TKA is associated with significantly greater intraoperative blood loss, longer operative times, and higher perioperative morbidity [5,6]. These factors highlight the need for effective, safe, and scalable hemostatic strategies. While the efficacy of ORC has been validated in unilateral TKA, its role in the more physiologically demanding setting of Sim B-TKA remains unclear.

The purpose of this retrospective study was to evaluate the effectiveness and safety of intraoperative ORC use in Sim B-TKA, expanding upon previous findings in unilateral TKA and focusing on postoperative hemoglobin (Hb) and hematocrit (Ht) levels, estimated blood loss (EBL), transfusion requirements, and related complications.

This manuscript is based on a study that was previously submitted in Japanese to the Journal of the Japanese Society for Replacement Arthroplasty. Permission for this English-language submission has been obtained from the journal.

## Materials and methods

This retrospective study included patients who underwent Sim B-TKA at Showa Medical University Fujigaoka Hospital, Yokohama, Japan, after obtaining approval from the Showa Medical University Research Ethics Review Board (approval number: 2025-0284; approval date: September 16, 2025). Patients were divided into two groups based on the intraoperative use of ORC: the ORC group (S group; n=21) and the non-ORC group (N group; n=28).

The primary outcome measures were Hb (g/dL) and Ht (%) levels, recorded at three time points: preoperatively, postoperative day 4, and postoperative day 7. To eliminate the influence of transfusion on Hb and Ht levels, patients who received perioperative blood transfusions were excluded from this analysis (final sample: S group (n=19) and N group (n=25)). EBL (mL) was calculated using the Gross formula [7] as follows: $\text{EBL}=\text{PBV}\times\left(\text{Hct\_pre}-\text{Hct\_post}\right)/\text{Hct\_avg}$. Here, PBV is patient blood volume (mL) which was calculated using Nadler et al.'s formula [8] based on patient sex, height, and weight, Hct_pre is preoperative hematocrit, Hct_post is postoperative hematocrit, and Hct_avg is the average of pre- and postoperative hematocrit values. All surgeries were performed under general anesthesia using a pneumatic tourniquet. The same standardized surgical protocol was applied in all cases, including a medial parapatellar approach, measured resection technique, and cemented fixation. Perioperative management, such as intravenous fluid replacement, anesthesia, and postoperative care, followed a uniform institutional protocol. Secondary outcomes included the incidence of postoperative complications related to ORC use and the transfusion rate. Continuous variables are expressed as mean±standard deviation (SD), and categorical variables are expressed as number (percentage). Group comparisons were performed using the Wilcoxon rank-sum test for continuous variables and the chi-squared test for categorical variables. All statistical tests were two-sided, and a p-value of <0.05 was considered statistically significant. Corresponding test statistics (Z or χ² values) were also reported. Data were analyzed using JMP® Pro version 16.0.0 (SAS Institute Inc., Cary, North Carolina, United States).

## Results

There were no significant differences in preoperative Hb and Ht levels between the ORC group (S group) and the non-ORC group (N group) (Hb: z=-0.01; p=0.99; Ht: z=-0.12; p=0.91; Wilcoxon rank-sum test). On postoperative day 4, the ORC group showed significantly higher Hb and Ht levels (Hb: 10.5±1.4 vs. 9.1±1.2; z=3.02; p=0.002; Ht: 31.6±3.8 vs. 27.7±3.4; z=3.12; p=0.002) and lower EBL (725.4±486.2 vs. 1188.7±546.2; z=-2.77; p=0.006). Similarly, on postoperative day 7, the ORC group maintained significantly higher Hb and Ht levels (Hb: 10.5±1.2 vs. 9.7±1.1; z=2.22; p=0.03; Ht: 31.7±3.1 vs. 29.4±3.1; z=2.38; p=0.02) and exhibited significantly lower EBL (686.1±416.9 vs. 970.6±481.1; z=-2.06; p=0.04). No statistically significant difference was observed in the transfusion rates between the groups (S group: 2/21 (9.5%); N group: 3/28 (10.7%); χ²=0.02; p=0.88). Additionally, no postoperative complications attributable to ORC use were observed in any patient (Tables 1-2).

**Table 1:** Comparison of patient demographics between groups Comparison of sex distribution, age, and BMI between the ORC (S group) and non-ORC (N group). Data are presented as mean±SD for continuous variables and as number (percentage) for categorical variables. Statistical tests used are the Wilcoxon rank-sum test for continuous variables and the chi-squared test for categorical variables. A p-value of <0.05 was considered statistically significant. Corresponding test statistics (Z or χ² values) are provided. ORC: oxidized regenerated cellulose; SD: standard deviation; BMI: body mass index; NA: not applicable (data not collected or not relevant for this group)

Variable	S group (ORC)	N group (non-ORC)	Test statistics	P-value
Number of cases	19	25	NA	NA
Sex (female/male)	15/4	21/3	χ²=1.56	0.21
Mean age (years)	76.7±6.9	75.2±8.6	z=0.72	0.47
Mean BMI (kg/m²)	24.3±3.4	26.5±3.5	z=-1.85	0.06

**Table 2:** Perioperative changes in Hb, Ht, and EBL Comparison of perioperative Hb, Ht, and EBL between the ORC group (S group) and the non-ORC group (N group) at each time point (preoperative, postoperative day 4, and postoperative day 7). Data are presented as mean±SD. Statistical analyses were performed using the Wilcoxon rank-sum test. Corresponding Z-values are shown. A p-value of <0.05 was considered statistically significant and is indicated with an asterisk (*). Hb: hemoglobin; Ht: hematocrit; EBL: estimated blood loss; SD: standard deviation; ORC: oxidized regenerated cellulose

Time point	Parameter	S group (ORC)	N group (non-ORC)	Test statistics	P-value
Preoperative	Hb (g/dL)	12.9±1.7	12.8±1.3	z=-0.01	0.99
	Ht (%)	38.8±4.1	38.5±3.2	z=-0.12	0.91
Post-op day 4	Hb (g/dL)	10.5±1.4	9.1±1.2	z=3.02	0.002*
	Ht (%)	31.6±3.8	27.7±3.4	z=3.12	0.002*
	EBL (mL)	725.4±486.2	1188.7±546.2	z=-2.77	0.006*
Post-op day 7	Hb (g/dL)	10.5±1.2	9.7±1.1	z=2.22	0.03*
	Ht (%)	31.7±3.1	29.4±3.1	z=2.38	0.02*
	EBL (mL)	686.1±416.9	970.6±481.1	z=-2.06	0.04*

## Discussion

Effective perioperative blood management remains critical in TKA, particularly in Sim B-TKA, which carries a higher risk of perioperative blood loss and thromboembolic complications than unilateral or staged procedures. Recent meta-analyses have consistently shown that Sim B-TKA increases the odds of pulmonary embolism, deep vein thrombosis (DVT), and short-term mortality, underscoring the importance of robust blood conservation strategies [9,10].

In this retrospective study of Sim B-TKA, intraoperative use of ORC was associated with reduced EBL and smaller postoperative declines in Hb and Ht, without increased transfusion requirements or complications. These findings are consistent with emerging randomized evidence in unilateral TKA. A 2023 RCT demonstrated that intra-articular ORC significantly reduced total and hidden blood loss compared with control, without a signal of harm [1]. Similarly, a 2025 single-blind RCT reported decreased EBL and attenuated postoperative thigh swelling with ORC versus placebo [11]. Collectively, these data support the hemostatic effectiveness and short-term safety of ORC in primary TKA.

Comparative data against TXA have also become available. A 2025 prospective RCT found that ORC and topical TXA achieved comparable reductions in total blood loss and Hb drop relative to control, suggesting that ORC is a reasonable alternative when TXA is unsuitable [2]. Beyond TKA, additional orthopedic studies, including hip arthroplasty, support ORC powder's hemostatic efficacy without worsening early outcomes, reinforcing its generalizability as a local mechanical hemostat [12].

TXA remains the cornerstone of pharmacologic blood management in arthroplasty, with extensive literature confirming its efficacy across administration routes [13,14]. However, caution or avoidance may be warranted in specific contexts such as high-dose seizure risk [15], renal impairment, or hypersensitivity. While some recent arthroplasty data suggest TXA is safe even in patients with a history of venous thromboembolism [16], careful patient selection and dosing remain important. In situations where TXA is contraindicated or undesirable, mechanical agents such as ORC provide a practical option.

There is also growing interest in combination strategies. A recent prospective, blinded RCT demonstrated that TXA combined with an absorbable hemostat reduced perioperative blood loss more effectively than either agent alone, without increasing thromboembolic or wound complications, suggesting potential additive benefit of pharmacologic and mechanical hemostasis in high-risk settings such as Sim B-TKA [17]. Although this trial did not exclusively evaluate ORC, the findings highlight the potential of multimodal protocols.

Safety considerations for ORC warrant attention. Although RCTs did not identify excess adverse events, rare hypersensitivity or foreign-body reactions have been reported following TKA with ORC powder [18]. Surgeons should apply the minimal effective amount, avoid intravascular use, and remain vigilant for local reactions.

This study has limitations. It was retrospective and single-institutional and lacked randomization, with blood loss estimated rather than directly measured. In addition, variations in surgical technique and perioperative management (e.g., anesthesia, fluid replacement, and tourniquet use) were not analyzed in detail, which may represent potential confounding factors influencing perioperative blood loss. Future studies with standardized operative and anesthetic protocols are warranted to minimize such bias. The modest sample size further restricts causal inference. Future multicenter prospective trials, ideally randomizing Sim B-TKA patients to ORC, TXA, their combination, or standard care, are warranted to define optimal protocols and to clarify patient subsets most likely to benefit.

In conclusion, intraoperative application of ORC appears to be a promising and safe hemostatic adjunct in Sim B-TKA, associated with reduced perioperative blood loss and postoperative anemia without increased complications.

## Conclusions

ORC demonstrated effective and safe hemostatic performance in Sim B-TKA, being associated with reduced perioperative blood loss and attenuated postoperative anemia without an increased risk of complications. ORC may serve as a valuable option within multimodal blood management strategies, particularly for high-risk procedures or in patients for whom TXA is contraindicated. Future large-scale, prospective trials are warranted to validate these findings and investigate the potential additive efficacy of combining ORC with pharmacological agents, such as TXA.
